# Conflagration from the Use of the Thermo-Cautery during Anæsthesia from Ether

**Published:** 1880-04

**Authors:** 


### CONFLAGRATION FROM THE USE OF THE THERMO-CAUTERY DURING ANÆSTHESIA FROM ETHER.

THE *British Medical Journal* (November 22, 1879,) from a French source, gives an account of an operation under ether for arthritis of the knee-joint, in which the actual cautery was employed. Five ounces of ether had been employed. The window had been opened, the room was large, and the ether-bag was to a certain extent separated from the thermo-cautery. Suddenly the room was in flames, and the bed was enveloped in them. The ether-bag was thrown down on the floor and the patient quickly removed. She was only slightly burned, but the physician who was administering the ether was severely injured. Similar accidents have been noticed elsewhere. Ignition does not occur when the wire is only heated to redness: a white head is necessary. Some years ago Dr. Dolbeau practiced local anæsthesia with ether spray on the hæmorrhoids of a patient about to be operated upon. The apparatus having been removed, the red-hot iron was applied, but the ether vapor caught fire, and produced a general conflagration and extensive burning of the surrounding parts without affecting the hæmorrhoids.

